# The effect of in vitro digestion on the interaction between polysaccharides derived from *Pleurotus eryngii* and intestinal mucus

**DOI:** 10.1002/fsn3.3845

**Published:** 2023-11-22

**Authors:** Sai Ma, Xinyi Li, Qi Tao, Qiuhui Hu, Wenjian Yang, Benard Muinde Kimatu, Gaoxing Ma

**Affiliations:** ^1^ Collaborative Innovation Center for Modern Grain Circulation and Safety, Jiangsu Province Engineering Research Center of Edible Fungus Preservation and Intensive Processing, College of Food Science and Engineering Nanjing University of Finance and Economics Nanjing China; ^2^ College of Food Science and Technology Nanjing Agricultural University Nanjing China; ^3^ Department of Dairy and Food Science and Technology Egerton University Egerton Kenya

**Keywords:** digestive products, interaction, intestinal mucus, *Pleurotus eryngii* polysaccharides

## Abstract

*Pleurotus eryngii* polysaccharides (PEPs) have been proven to display multiple activities through digestive system action, from which the digestion products should first interact with intestinal mucus (MUC), followed by the function of intestinal cells. Hence, possible interacting characterizations between MUC and in vitro simulated digestion products of *P. eryngii* polysaccharides (DPEPs) and PEP were carried out in the present study. Results showed that both PEP and DPEP could significantly interact with MUC. Moreover, digestion can modify the interaction between polysaccharides and MUC; the degree of interaction also changes with time incrementing. Viscosity could be decreased after digesting. According to the zeta potential and stability analysis result, the digestive behavior could be regular and stable between polysaccharides and MUC interactions. Following fluorescence and infrared spectra, the structure of polysaccharides and mucin might be changed by digestion between polysaccharides and MUC. The study indicates that the interaction formed between DPEP and MUC might indirectly impact the exercise and immune activities of polysaccharides and influence the transportation of other nutrients. Overall, our results, the absorption and transport pathways of PEP, can be initially revealed and may provide a novel research viewpoint on the active mechanism of PEP in the intestinal tract.

## Graphical abstract

This study proved that digestion products of *P. eryngii* polysaccharides and *P. eryngii* polysaccharides could interact with intestinal mucus. Moreover, digestion can change the interaction between polysaccharides and intestinal mucus and the degree also changes with time increasing between polysaccharides and intestinal mucus interaction.

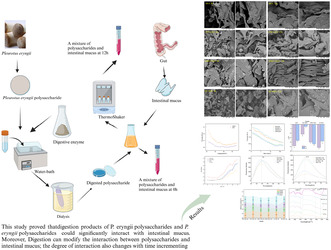

## 1 INTRODUCTION

The gastrointestinal tract is often considered an essential organ involved in the digestion of food and providing nutrients to the body. This environment of the gastrointestinal tract is composed of contents extremely complex. However, intestinal mucus (MUC) in the intestinal tract could effectively prevent pathogenic bacteria, endotoxins, and other harmful substances from crossing the intestinal mucus to the blood and the abdominal cavity (Jia & Han, 2023; Lai et al., 2022). Only the digestion products of *Pleurotus eryngii* polysaccharides (DPEPs) through MUC can exert activities by stimulating intestinal cells. Nevertheless, whether digestive polysaccharides interact with MUC and how this interaction affects the functional activity of polysaccharides are less studied at this stage (Pham & Nöjd, 2021) reported mucin carrying intrinsic cross‐linker residues (e.g., disulfide bonds) and reactive functional groups (e.g., sialic acid), hence providing many opportunities for interactions with the MUC (Liu & Yu, 2020).

After polysaccharides enter the intestine, polysaccharides could adhere to the MUC to form polysaccharides–intestinal mucus complexes. Research shows that at high concentrations, the interaction of chitosan and mucin in vivo alters the attachment of the biopolymer to the mucosal surfaces and forms entangled and gel networks (Collado‐González & González Espinosa, 2019). Zhang & Cheng (2018) indicated that not only mucoadhesion is essential to achieve the bioavailability of the drugs transported but also that the ability of the structures to penetrate the mucus plays a vital role in the permeation efficiency. An investigation has shown that pH‐dependent mucin–procyanidin interactions were disrupted by the addition of ethanol and dimethylsulfoxide (DMSO) (Brandão & Santos Silva, 2017). Adding polyphenols changes the mucin solution's physical properties and alters the degree of cross‐linking of the solution (Georgiades & Pudney, 2014a, 2014b). Therefore, most studies have focused on the interaction between macromolecules and other substances and MUC and less on the mechanism of action between *P. eryngii* polysaccharides and MUC. Because of its multiple activities, such as hepatoprotective (Chen & Mao, 2012), antioxidant (Petraglia & Latronico, 2023), antitumor (Christodoulou & Vlassopoulou, 2023), lipid accumulation inhibitor (Chen & Yong, 2014), and anti‐inflammatory (Qi et al., 2023), *P. eryngii* has been industrially cultivated as a new type of cherished edible mushroom (Zhang & Song, 2021). Among them, PEP has garnered significant attention as active compounds among researchers. A study has shown that the interaction between the substances is closely linked to their physicochemical properties (Aljabali, 2023). Although PEP cannot be digested by the human body (Ma & Xu, 2022), alterations in their degree of polymerization and intermolecular connections may occur (Li & Xiong, 2016), consequently affecting the extent of interaction with MUC.

In the previous period, our team prepared a novel polysaccharide of *P. eryngii* and studies on structural characterization of polysaccharides and other related mechanisms have been carried out (The UV spectra and FTIR spectra of PEP are provided in SM, Figures S1 and S2, respectively). Meanwhile, the primary structure was comprehensively analyzed, encompassing determination of molecular weight, monosaccharide composition (Ma & Kimatu, 2020; Ma & Xu, 2022). But as the work progressed, the importance of the interaction between polysaccharides and MUC for their absorption and transport was recognized. This work aims to investigate the molecular‐level interactions between MUC and polysaccharides. These interactions are to be mapped as macroscopic phenomena. The phenomena are zeta potential, SEM, turbidity test, and stability analysis; then, the molecular‐level interactions of the mixture's components are deeply studied, which are to be linked to microscopic forces by fluorescence spectroscopy and Fourier transform infrared (FTIR). It was shown that polysaccharides can interact with MUC and that this effect is more pronounced in digested polysaccharides. More interestingly, this action becomes stable with time increasing 12 h. The study's results lay the initial analysis of their interaction characteristics with MUC and the indirect estimation of the pathways through which polysaccharides exert their immune activity in the intestine.

## 2 MATERIALS AND METHODS

### 2.1 Materials

*Pleurotus eryngii* powder was bought at Xinghua Lianfu Food Co. Pork intestine from Nanjing Xuanwu District Farmers' Market. α‐amylase, pepsin, and trypsin was purchased from Solarbio Science & Technology Co., Ltd. (Beijing, China). A protease inhibitor Cocktail was obtained from USA MedChemExpress Corporation (Bio). All other chemical reagents were of analytical purity.

### 2.2 Preparation of polysaccharides from *P. eryngii*

Preparation of polysaccharides from *P. eryngii* was done according to the method by (Ma & Xu, 2022). Briefly, *P. eryngii* powder (120 g) was soaked overnight in ethanol (85%) for decolorizing and degreasing. The mixture was then stirred and centrifuged (6400 × *g* for 20 min). The precipitate was mixed with ultrapure water (1:25 w/v) and heated at 60°C for 3.5 h with constant stirring, which was centrifuged (6400 × *g* for 20 min). The obtained supernatant was concentrated in a rotary evaporator before precipitating overnight with a 1:4 volume of absolute ethanol. The suspended solids were collected through centrifugation (4000 × *g* for 15 min), and the obtained pellet was re‐dissolved in ultrapure water and freeze‐dried to receive PEP.

### 2.3 The production of digestion polysaccharides (DPEPs)

A 9 mg/mL solution of PEP was taken and subjected to three stages of the in vitro human digestion model system (Brodkorb & Egger, 2019), representing the oral, gastric, and intestinal stages of digestion.

First digestion phase (oral phase): first, the digestion solution was mixed with PEP solution (0.3%) at a 1:1 ratio, then the mixture was stirred for 2 min at 37°C with a pH of 7.0 to simulate oral conditions.

Second digestion stage (gastric phase): operated at a solution with a pH of 3.0 ± 0.1, the gastric digestion solution was added to the digestion chamber configured in the first stage at a ratio of 1:1 and stirred for 2 h at 37°C.

Third digestion stage (intestinal phase): operated under a solution of a pH value of 7.0 ± 0.1, the intestinal stage digestion solution was configured in a ratio of 1:1 added to the second digestion solution and stirred for 2 h at 37°C.

After preparation, the insoluble enzyme was removed by centrifugation, and the supernatant was taken for dialysis to remove ions and small molecules. Finally, the dialysate was concentrated in a rotary evaporator, freeze‐dried into a powder, and stored in a drying chamber for the next experiment.

To reduce the error caused by digestive enzymes, control groups (DJ + MUC 0 h, DJ + MUC 12 h) were set. The other groups were arranged as follows: mixing of *P. eryngii* polysaccharide with IM (PEP + MUC 0 h, PEP + MUC12h); mixing of digested *P. eryngii* polysaccharide with intestinal mucus (DFPEP + MUC 0 h, DEPEP + MUC12h).

Saliva digestive juice (SSF), gastric digestive juice (SGF), and intestinal digestive juice (SIF) were prepared in advance. The proportions of the prepared solutions are provided in Table 1.

**TABLE. 1:** Simulation chylous preparation parameters.

Reagent	Concentration (Mol/L)	SSF (mL)	SGF (mL)	SIF (mL)	Enzyme activity (μ/mL)
KCl	0.5	15.1	6.9	6.8	
KH_2_PO_4_	0.5	3.7	0.9	0.8	
NaHCO_3_	1	6.8	12.5	42.5	
NaCl	2	‐	11.8	9.6	
MgCl(H_2_O)_6_	0.15	0.5	0.4	1.1	
(NH_4_)_2_CO_3_	0.5	0.06	0.5	–	
Alpha‐salivary amylase					75 μ/mL
Pepsin					2000 μ/mL
Trypsin					100 μ/mL

*Note*: The final volume of each simulated fluid is 500 mL.

### 2.4 Intestinal mucus extraction

Pig intestinal mucus was extracted by referring to Mackie and Macierzanka (2016). method. Fresh pig intestines were cut into small sections, washed with deionized water, and then washed with 0.01 mol/L phosphate buffered solution (PBS) (8 g/L NaCl, 0.2 g/L KCl, 3.63 g/L NaH_2_PO_4_–12H_2_O, 0.24 g/L KH_2_PO_4_) to clean the intestinal debris and maintain intestinal pH balance of MUC. After discarding the PBS, the intestine was rubbed by hand until the mucus appeared on the surface, which was then scraped off and carefully collected. A protease inhibitor Cocktail was added before storing at −80°C to inhibit protein phosphorylation or other effects of proteases.

### 2.5 Measurement of rheology of polysaccharides

#### 2.5.1 Effect of temperature on the viscosity of *P. Eryngii* polysaccharides

The viscosity of PEP and DPEP solutions at different temperatures was measured using an MCR3302 rheometer. The samples were dissolved in deionized water and prepared into a solution with a concentration of 0.3%. The samples were added to a rheometer plate (50 mm diameter, 100 μm gap) at temperatures ranging from 25°C to 90°C. The variation of the viscosity of the samples with temperature was examined, and the temperature was measured by adding a seal and a drop of methyl silicone oil during the experiment to avoid excessive water evaporation. For each sample, the experiment was repeated three times.

#### 2.5.2 Effect of shear rheology on viscosity of polysaccharide

The viscosity of polysaccharide solutions at different pH was investigated using MCR3302 rheometer. PEP and DPEP samples were dissolved in deionized water to a concentration of 0.3%. The pH of PEP and DPEP samples was adjusted respectively to 1.5 and 8.0, using 1 mol/L HCl and 1 mol/L NaCl. The samples were added drop‐wise to a rheometer plate (50 mm diameter, 100 μm gap) at a temperature of 37°C and a shear rate of 0.1 ~ 200 s^−1^, and the changes in the viscosity of the samples at different pH values were measured in three parallel experiments.

### 2.6 Zeta potential

The undigested and control groups (digestive enzyme freeze‐dried powder) were separately mixed with an appropriate amount of freeze‐dried powder in MUC to prepare a 0.3% polysaccharide solution. The DPEP group's solution was prepared as follows: Since the actual content of DPEP is obtained by subtracting the digestive enzyme powder from the total freeze‐dried powder, a 3 mg/mL concentration of MUC and polysaccharide solution was prepared based on the ratio of actual DPEP powder to the total freeze‐dried powder. The following solutions are configured in this proportion. Based on a previous study and modification (Crudden & Afoufa‐Bastien, 2005), the polysaccharide and MUC mixtures were divided into two portions for 0 and 12 h, where the 0 h measurements were taken directly and the 12 h were measured after being placed in a bench‐top thermostatic shaker at 37°C until 12 h. The zeta potential of the mixture of polysaccharides MUC was measured at 37°C for 0 and 12 h. A sample of 1 mL of the mixed solution was taken in a cuvette and then equilibrated in Nano ZS90 for 2 min, and nine continuous light scattering readings were taken. The experiment was repeated three times.

### 2.7 Scanning electron microscopy

The mixed solution containing 0.3% polysaccharides and MUC was poured into a Petri dish, incubated in a thermostatic shaking chamber for 0 and 12 h, and freeze‐dried in vacuo. The dried sample powder was then placed on a double‐sided conductive metal dish and coated with a gold layer. The surface morphology and microstructure of the samples at 0 and 12 h were observed with a TM3000 scanning electron microscope (SEM) (Gao & Zhang, 2018).

### 2.8 Turbidity analysis

Referring and modifying the method by Souza and Garcia‐Rojas (2015), the PEP + MUC, DPEP + MUC, and DJ + MUC solutions were added into 12 96‐well plates at 200 μl per well volume respectively (each 96‐well plates contains each sample and set 6 repeated holes for each sample) and placed in a temperature‐controlled oscillator set at 37°C then incubation for 12 h. The optical density (OD) values were measured at a wavelength of 600 nm every hour until all the wells in the 12 plates were completed.

### 2.9 Stability analysis

Four milliliter of MUC sample was added to the two sample bottles of the multiple light scattering instrument (TURBISCAN Lab), and after the liquid surface was leveled, 16 mL of PEP, DPEP, and digestive juice (DJ) concentrated in 0.3% solution were slowly added. Parameter settings: test temperature 37°C, scanning frequency 5 min scanning once, total 12 h scanning (Salleh & Goh, 2022).

### 2.10 Fluorescence spectrum

The alterations in the tertiary structure of PEP and mucins were investigated by fluorescence spectroscopy. The final concentration of 0.3% polysaccharide solution was configured. According to a reference with some modifications (Chang & Wang, 2017), the excitation wavelength was set at 290 nm (λ_ex_), and the emission signal was gathered in the range of 300–500 nm (λ_em_). The slit width was 5 nm, and the scanning speed was 1200 nm/min.

### 2.11 Fourier infrared spectroscopy measurements

According to a description with some modifications (Chang & Wang, 2017), 1 mg of sample powder and 100 mg of dried potassium bromide powder were taken and placed in an agate mortar and ground simultaneously. Then, the ground powder was transferred to the tablet press mold with a small stainless steel flat spatula and spread out to ensure a uniform and transparent ingot. Finally, the entire mold was placed in the press, and a force of about 20 KPa was applied for about 2 min; afterward the pressed ingots were removed and then scanned in the wave number range of 4000–400 cm^−1^ at a frequency of 16 and a resolution of 4.

### 2.12 Data analysis

Origin 9.0 software was used to plot the infrared data, Omnic and Peakfit were used to process the infrared data, and TurbiscanLab, the special analysis software for the stability analyzer, was used for stability analysis. SPSS software was applied to perform one‐way ANOVA with *p* < .05 as the test of significance.

## 3 RESULTS AND DISCUSSION

### 3.1 Effect of in vitro digestion on the viscosity of polysaccharides

#### 3.1.1 Effect of temperature on viscosity of polysaccharide

**FIGURE 1:**
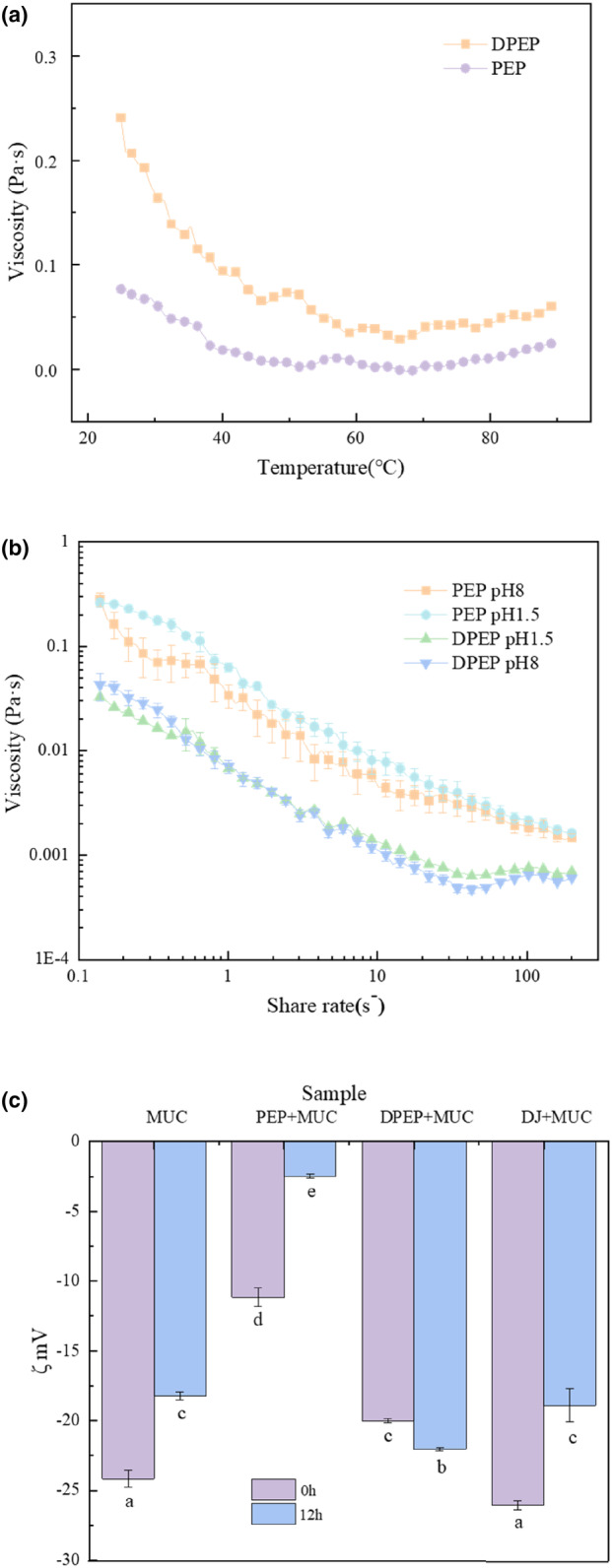
Physical indexes affecting the interaction between intestinal mucus and different samples. (a) Effect of temperature on viscosity; (b) polysaccharide viscosity shear curve; (c) zeta‐potential values of polysaccharides and intestinal mucus. The values with small superscript letters indicate significant group differences, respectively (*p* < .05).

In order to preliminarily predict the interactions between polysaccharides and MUC, it is imperative to explore the viscosity of polysaccharides to study their adhesion behavior within the intestinal. The investigation of temperature's impact on the viscosity of polysaccharides provides valuable insights into alterations in their intermolecular interaction forces. Figure 1a shows that the viscosity of the polysaccharides solution decreased as the temperature enhanced, and PEP viscosity was greater than that of the digestive products of PEP. Studies have shown that the viscosity of polysaccharides showed a negative relationship with the temperature (Tao & Ma, 2022). Indeed, the increase in temperature could intensify the thermal movement of molecules, break the hydrogen bonds, and weaken the entanglement between macromolecules, resulting in the “dilution effect,” which causes the apparent viscosity of polysaccharides to decrease (Anvari & Tabarsa, 2016; Gallão et al., 2013). This study's finding suggests that digestion can lead to a reduction in the viscosity of polysaccharides. It seems that makes it easier to pass through to MUC, which serves as a molecular sieve.

#### 3.1.2 Effect of shear rheology on viscosity of polysaccharide

Measurements of viscosity versus shear rate can be used to provide information about the strength of macromolecule interaction (Fernandez‐Avila et al., 2015). Previous studies have shown that the increased viscosity of psyllium husk slows gastric emptying (Bergmann & Chassany, 1992). Therefore, viscosity affects the residence time of substances in the intestines. Thus, this experiment explores the influence of shear force on polysaccharide viscosity, which can also simulate the chewing of polysaccharides in the oral cavity. At the same time, the investigation of viscosity changes at different pH values was conducted through the simulation of gastrointestinal pH. As Figure 1b shows, two typical shear‐thinning behavior curves are observed. The viscosity of the polysaccharides all decreased with increasing shear rate, and the digestion products were more pronounced for DPEP than for PEP. Interestingly, from this figure, the pH effect on the viscosity of DPEP is little, but the viscosity of PEP is greater in acidic conditions than in alkaline. The digestion process can effectively decrease the viscosity of polysaccharides as a whole and alter the charge state of polysaccharides. It is evident that the level of interaction between polysaccharides and MUC is changed due to digestion. Most investigations have demonstrated that polysaccharides are not broken down by enzymes in the human host (Ma & Xu, 2022), yet the structure and intermolecular covalent bonds of the agglomerated polysaccharides opened or changed by digestion, and the intermolecular interaction is reduced, which is the reason why with the same shear rate the viscosity changes of PEP and DPEP are different. It is important to consider that the reason the viscosity is higher in acidic conditions than in alkaline conditions is that the intermolecular network structure is strengthened, and new connections are established by hydrogen bonding (Sosnik & das Neves, 2014). On the other hand, the electrostatic repulsive forces and negative charge density are reduced under acidic conditions due to the interaction of hydrogen ions with the unlinked groups in the molecular helix structure (Kani & Horinaka, 2005; Yamamoto, 2007). Furthermore, an increase in viscosity is obtained when more electrostatic forces exist intermolecular. When the electrostatic repulsive forces drop, the spiral structure of polysaccharides compresses and aggregates, causing the motion of the chain to be limited (Lieleg & Vladescu, 2010). Hence, next, we will continue to explore the interaction characteristics by measuring the zeta potential of substances.

### 3.2 Determination of zeta potential

Electrostatic forces may occur in the interaction between polysaccharides and MUC (Picos‐Corrales et al., 2023), involving patches of positive charge such as arginine, histidine, and lysine in the mucin protein backbone, as previously discussed in the case of alginate–mucin interaction (Nordgard, 2011). At the same time, protein–polysaccharide interactions are mainly controlled by electrostatic forces (Klein & Aserin, 2010; Niu & Su, 2014). To consider this further, we must examine the nature of the electrostatic interaction between MUC and polysaccharides (Menchicchi & Fuenzalida, 2015).

Figure 1c shows that compared with PEP + MUC group (−11.17 ± 0.66 mV, −2.48 ± 0.15 mV), the ζ‐potential of DPEP + MUC group (−20 ± 0.14 mV, −22.03 ± 0.12 mV) increased. Surprisingly, with the increase in time, except for the zeta potential of the DPEP + MUC group rise, the other groups decreased. According to the bar chart of MUC and DJ + MUC, there was little difference in potential between the two groups, indicating that DJs had almost no effect on the interaction between polysaccharides and MUC.

The results show that polysaccharides can interact with MUC and that digestion can change the electrostatic interaction between polysaccharides and MUC, and the electrostatic repulsion in DPEP + MUC is dominant over time. This means that after polysaccharide digestion, the molecular distribution of the polysaccharide MUC system is more uniform than that in PEP + MUC group and has an increasing trend with time. Because digestion changes the structure and total molecular weight of polysaccharide molecules, the interaction system changes after digestion, consistent with the interaction between deacetylated chitosan and MUC (Morris & Nishinari, 2012). Also, it is possible that the molecular agglomerative structure of the polysaccharides was opened after digestion, resulting in steric resistance among intermolecular (Yuan & Ritzoulis, 2019). It is speculated that the more considerable absolute value of DPEP + MUC potential could be that digestion increases the effective specific surface area of the structure of the polysaccharides, causing an increased probability of other molecules being adsorbed (Limbach, 2005). Moreover, previous studies have shown that the higher the absolute value of zeta potential, the higher the electrostatic repulsive force among nanoparticles, which could improve the stability of nanoparticles (Chen & Ma, 2021). This is consistent with the uniformity of our results. Electrostatic interactions can affect particle formation. Therefore, we will further observe the macroscopic effect of digestion on the interaction between polysaccharides and MUC.

### 3.3 Scanning electron microscopy (SEM)

To better understand the effect of digestion on the interaction between polysaccharides and MUC, the lyophilized droplets of polysaccharides–intestinal mucus mixtures were observed by scanning electron microscopy in this study.

**FIGURE 2:**
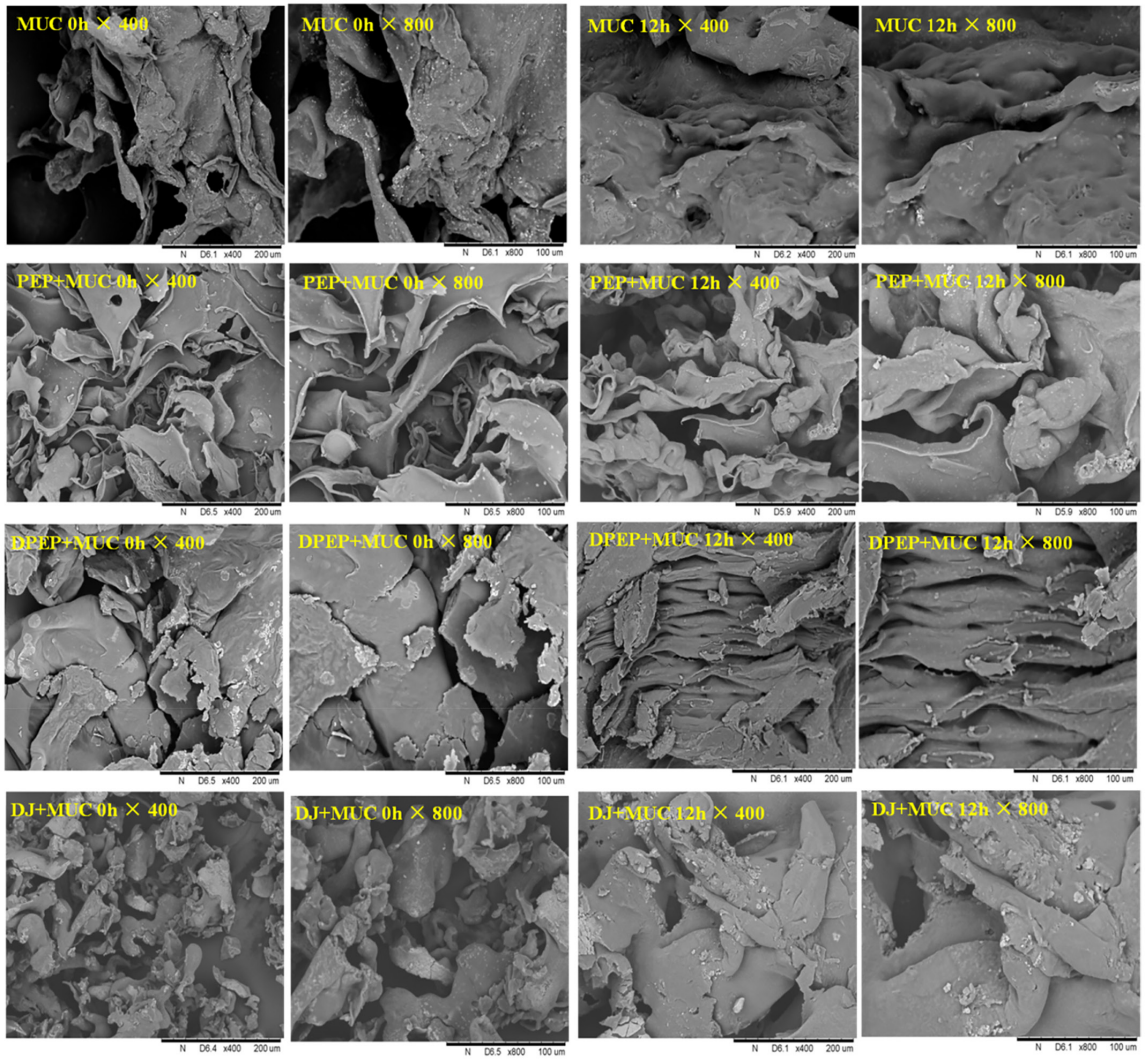
SEM images of complexes of intestinal mucus and different samples spray‐dried.

As depicted in Figure 2, both a comparison between PEP + MUC 0 h/12 h and DPEP + MUC 0 h/12 h reveals that PEP + MUC 0 h/12 h predominantly exhibits sheet folds with a porous network, while the latter demonstrates a blocky structure with enhanced material adhesion, resulting in thicker and stronger cohesion. Furthermore, observations of PEP + MUC group at different times indicate the sheet folds have significant thickening accompanied by reduced gap at 12 h, leading to an enlarged pore size. Similarly, DPEP + MUC group's 12 h structure appears more regular, cohesive, and entangled in the network. Both MUC and DJ + MUC exhibit temporal trends of expansion and condensation. The structure of PEP + MUC and DPEP + MUC is obviously different from that of MUC. The results show that polysaccharides can interact with MUC, and digestion can change the interaction between polysaccharides and MUC. In a deeper view, as the interaction time prolongs, it was possible to observe an entangled, interconnected three‐dimensional network that can be observed to be gradually “ filled.” After digestion, the random coiling of the polysaccharides is modified into a helical conformation that gradually binds to MUC with time increasing, forming larger molecules (Picone, 2011). However, the specific changes after they pass through the intestinal mucus layer and before they exert their activity are unknown in polysaccharides, so it is vital to explore this interaction to obtain a deeper understanding of the indirect activity of polysaccharides.

### 3.4 Turbidity test

To determine the effect of digestion on the interaction between polysaccharides and MUC and to reflect the dispersion of the cohesive state of the complex, the turbidity of the mixture of polysaccharides and MUC was investigated within 0–12 h.

**FIGURE 3:**
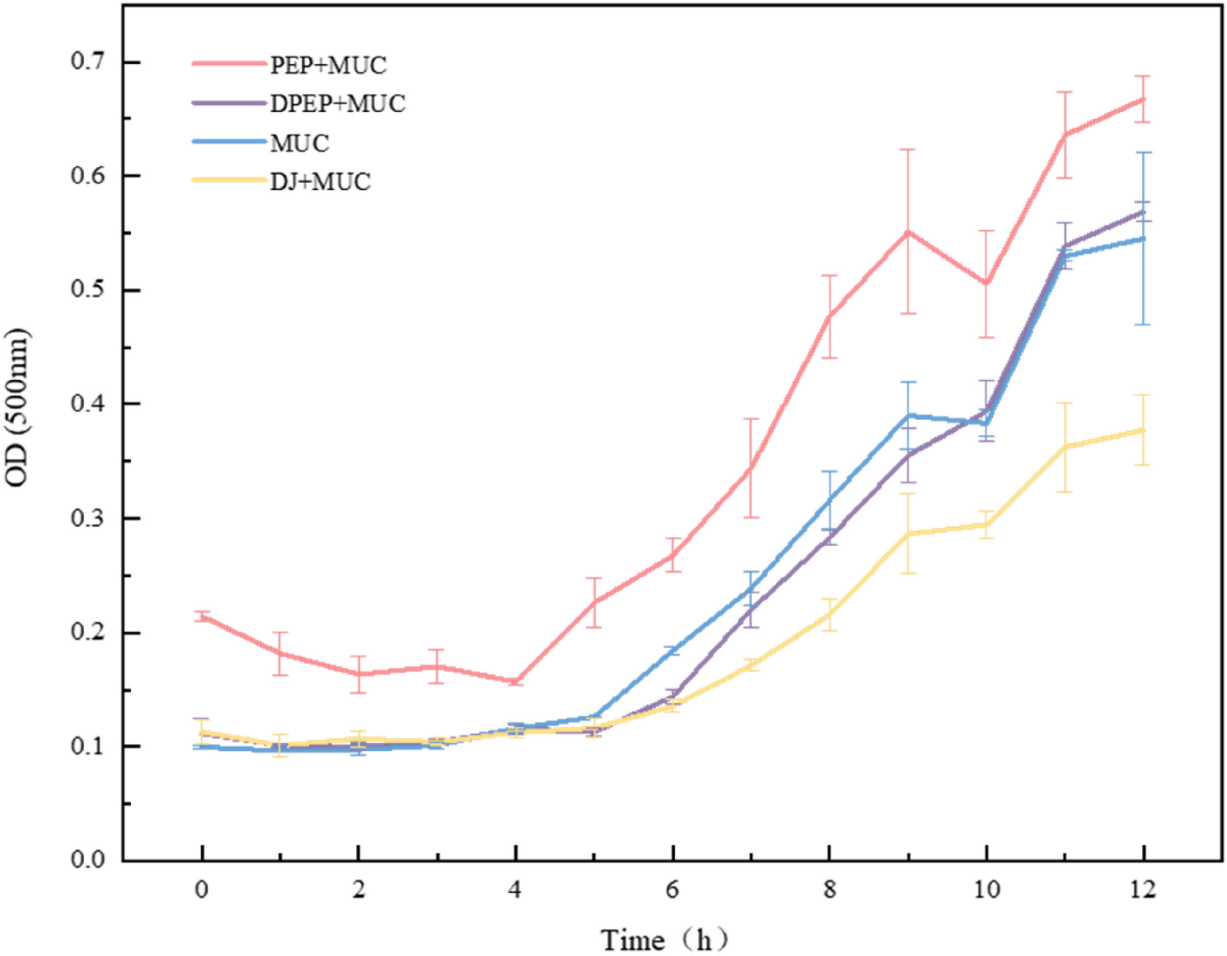
Turbidity variation curves of different samples in 0–12 h.

Figure 3 shows that polysaccharides can interact with MUC and that digestion affects polysaccharides–intestinal mucus interactions. DPEP and MUC form a stable dispersion in the solution, slightly causing particle aggregation. Meanwhile, from 0 h, the turbidity of the other mixed solution system increased as time increased. Surprisingly, the group that added MUC had a significant tendency to a greater rate of particle aggregation after 5 h. The phenomenon could be attributed to the agglomerative structure of polysaccharides by digestion, resulting in the complex formed after binding with MUC being more soluble in water. At this time, the turbidity reduction could be explained as the flocculation of protein–polysaccharides in the system, and the molecules will all be distributed in a stable state in the solution, causing the diffuse scattering of the solution to be reduced. Weinberg et al. also proved that electrostatic interactions can aggregate proteins and polysaccharides and that the resulting complex condensates affects turbidity (Weinbreck, 2003). The exerting of intestinal barrier function tends to be more toward small‐scale particles, so studying the size of the particles formed by polysaccharides and MUC is necessary to deeply investigate how polysaccharides are active in the intestine (Mackie & Macierzanka, 2016).

### 3.5 Stability analysis

**FIGURE 4:**
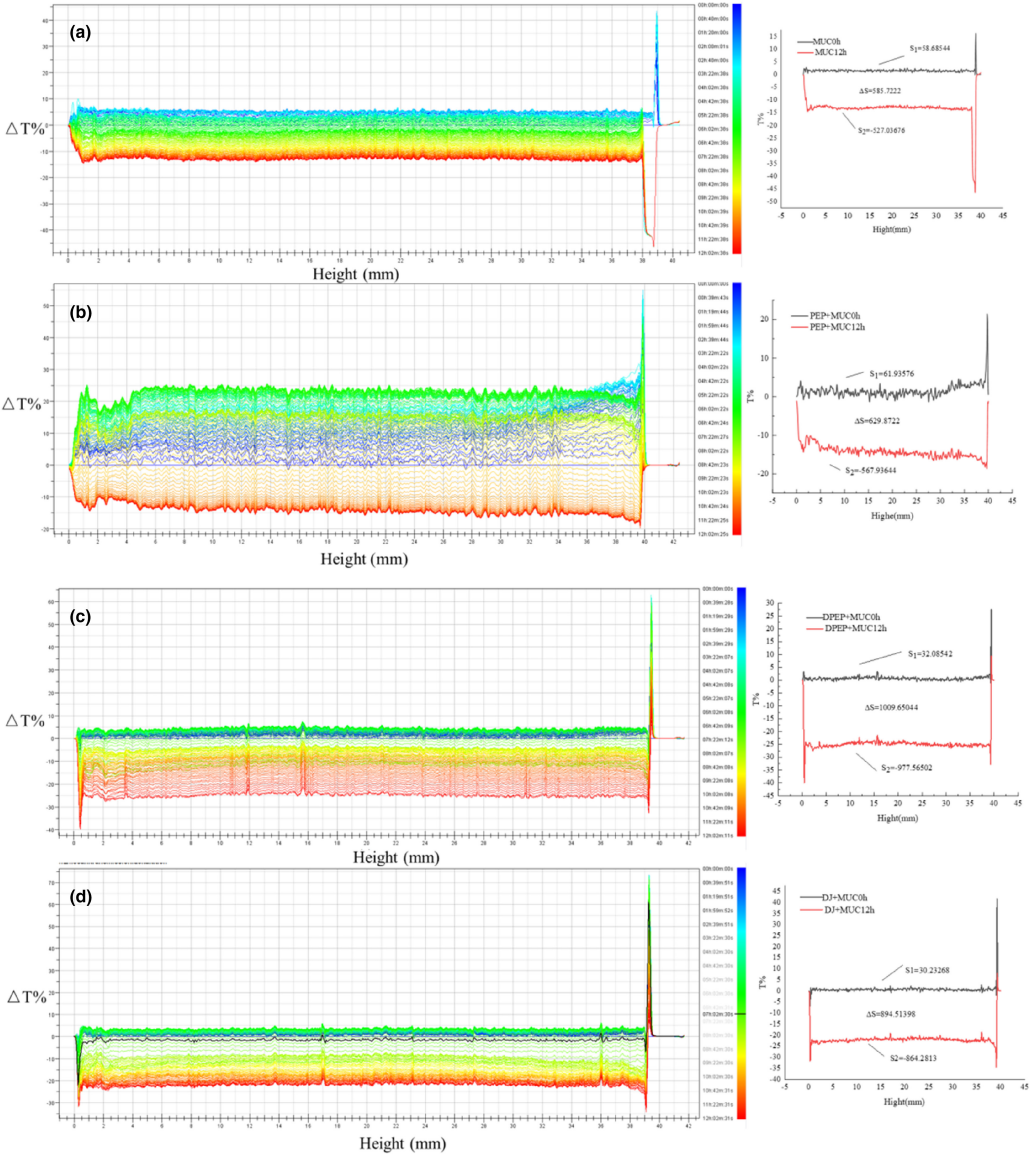
Transmission profiles of MUC (a), PEP + MUC (b), DPEP + MUC (c), and DJ + MUC (d).

To better understand the interaction between polysaccharides and MUC, a TurbiscanLab multi‐light reflection analyzer was used to examine the dispersion system of polysaccharides and MUC in real time (Bordes & Snabre, 2003). That is, scanned the precipitation and clarification process of polysaccharides and MUC particles within 12 h. Figure 4 suggests that digestion can cause changes in the interaction between polysaccharides and MUC. Figure 4a shows the stability analysis graph of MUC; the intensity of transmitted light at the bottom of 0–1.5 mm increases with time while the intensity of transmitted light at 5–12 h decreases. Figure 4b,c shows the stability analysis of PEP and DPEP with MUC mixture; according to Figure 4b,c, it can be seen that ΔT% at the bottom of PEP + MUC (0–1.6 mm) and DPEP + MUC (0–1.25 mm) settles faster and accumulates more particles as time increases, which is consistent with the turbidity analysis. The transmitted light intensity at the top of PEP + MUC and DPEP + MUC, such as 39.5–40 mm and 39.25–40 mm with time increasing, will decrease when reaching a specific time point. Figure 4d shows digestive juices (DJ) mixed with MUC, and it is observed that the trend closely resembles that of DPEP mixed with MUC, but the curve decreases more regularly. Under the same conditions, the transmitted light in the upper middle of the solution of the PEP + MUC group is stronger than the solution of the DPEP + MUC group, indicating that DPEP interacting with the MUC can be stably dispersed in the solution. There may be two reasons for this: first, the polysaccharides have a large molecular weight and high viscosity, making unstable aggregation with MUC easier. Second, the network structure of MUC changes time extending, and the mucin aggregates, causing changes in the mucin interaction with polysaccharides. Taken together, the interaction between polysaccharide–intestinal mucus is influenced by their physical and chemical properties, while digestion can alter its molecular structure and physical and chemical characteristics. It should be noted that this structural change can impact the exerting indirect activity of polysaccharides.

### 3.6 Fluorescence spectroscopy

To obtain further information on the interactions of polysaccharides and MUC, the fluorescence intensity of polymers was analyzed by fluorescence spectrum. It was reported that the intrinsic fluorescence of proteins is exhibited because of the presence of tryptophan (Trp), tyrosine (Tyr), and phenylalanine (Phe) (Ahmad & Ritzoulis, 2020).

**FIGURE 5:**
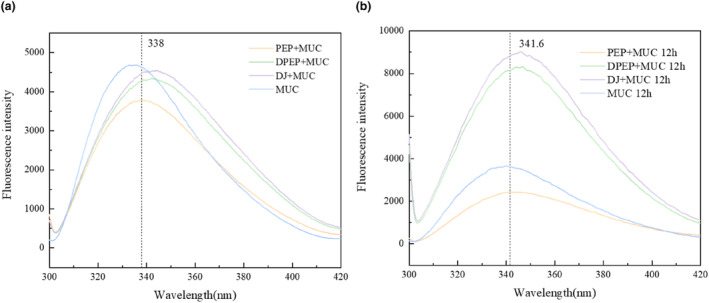
Fluorescence spectrum of different samples; Fluorescence spectrum of the sample at 0 h (a); Fluorescence spectrum of the sample at 12 h (b).

As depicted in Figure 5a,b, the comparison between PEP + MUC (338 and 341.6 are the positions of λ_max_ at 0 and 12 h, respectively) and DPEP + MUC (342.6 and 346 are the positions of λ_max_ at 0 and 12 h, respectively) shows a red shift in the maximum peak value of fluorescence intensity. Interestingly, the position of λ_max_ of both PEP + MUC (338 and 341.6 are the positions of λ_max_ at 0 and 12 h, respectively) and DPEP + MUC (342.6 and 346 are the positions of λ_max_ at 0 and 12 h, respectively) was altered due to time‐extending interaction with MUC. The prolonged interaction between polysaccharides and intestinal mucus leads to an intensified fluorescence intensity, a phenomenon that is particularly evident in the presence of digestion. These findings demonstrate that polysaccharide–intestinal mucus interactions can induce conformational changes in mucin's three‐dimensional structure, and the results are further influenced by digestion. Consequently, the fluorophore was exposed because of mucin's three‐dimensional structure unfolding during the interaction. At the same time, Sun's research shows that hyperchromism is related to unfolding protein folding (Sun & Dai, 2016). The interconnection between polysaccharides and mucin via Trp residues results in a decline in fluorescence intensity. However, interactions between DPEP and mucins may break hydrogen bonds in complementary regions, then cause charge repulsion between the chains, exposing the inherently fluorescent part. Moreover, this process extends the tri‐helical structure of mucin and induces the mucin molecules to be rearranged by polyanions (Ahmad & Ritzoulis, 2020; Menchicchi & Fuenzalida, 2015). In summary, the interaction between polysaccharides and MUC in vitro can change the tertiary structure of mucin, which has important significance for the absorption of intestinal flora and other nutrient substances.

### 3.7 Fourier transform infrared (FTIR)

FTIR spectroscopy is a valuable tool to analyze the secondary structures of proteins, in which the vibration of the peptide bonds in proteins, namely the amide I band is present at 1600–1700 cm^−1^ (Wang, 2011). Specifically, the bands at 1600–1640 cm^−1^, 1640–1650 cm^−1^,1650–1660 cm^−1^, and 1660–1700 cm^−1^ indicated the presence of β‐sheet, random coil, α‐helix, and β‐turn, respectively. Compared to 0 h, it was found that at 12 h the O‐H absorption peak of the polysaccharides interaction with MUC was significantly blue shifted (3395.88 cm−1, 3297.74 cm−1 are the positions of the absorption peaks at 0 and 12 h for the PEP + MUC group; 3385.4 cm−1, 3290.5 cm−1 are the positions of the absorption peaks at 0 and 12 h for the DPEP + MUC group). Furthermore, significant changes in the positions and shapes of infrared spectral bands for each component are observed. Notably, there is a distinct red shift at the position of amide I, especially at 12 h, while in the digestion group, the peak of amide II disappears. These findings indicate that the hydrogen bonding forces between digested polysaccharides and MUC are weakened, and there are changes in hydrophobic interactions, resulting in alterations in the secondary structure of mucin and polysaccharides. This demonstrates that digestion may lead to significant interactions (Zhou & Wang, 2016). Interestingly, the interaction between polysaccharides and MUC can induce alterations in the secondary structure of mucin. Similarly, digestion indirectly impacts the secondary structure of mucin by modulating their interplay.

**FIGURE 6:**
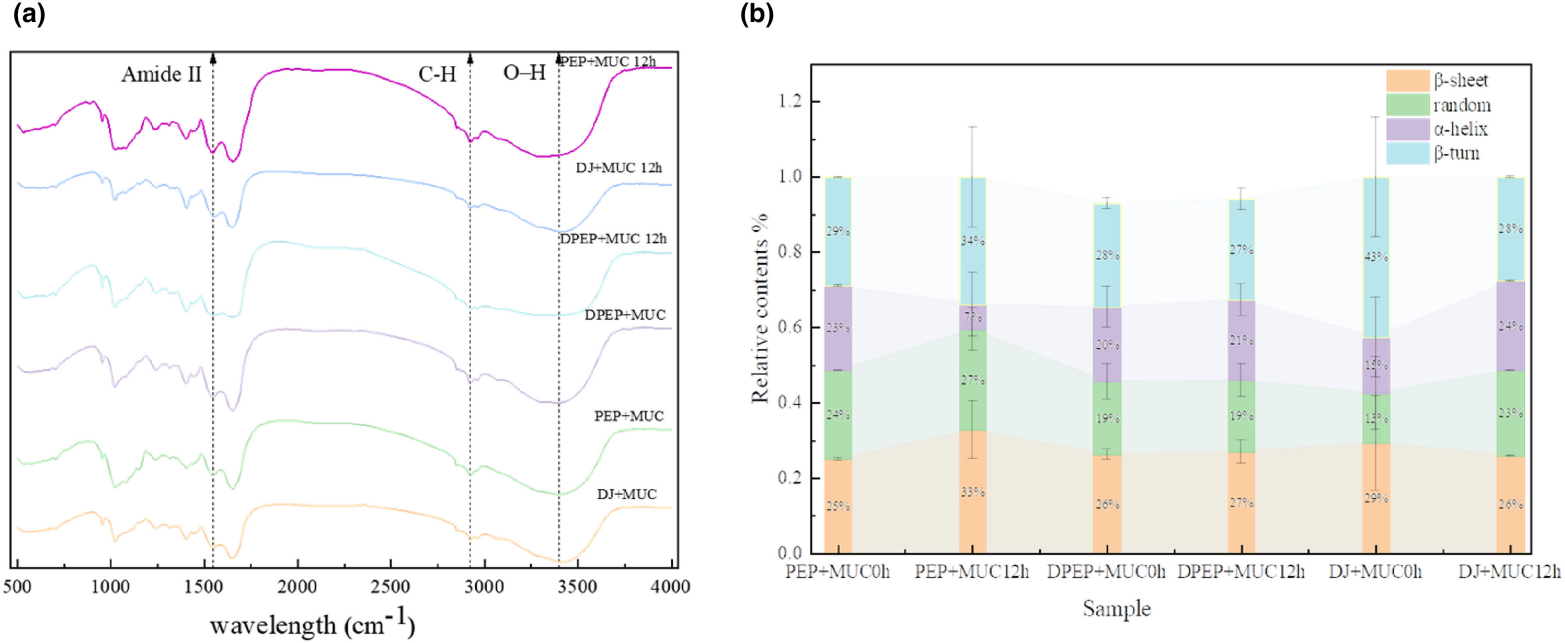
FTIR absorption behavior for the different samples (a) and the variation of the content of secondary structure (b).

Figure 6b shows the secondary structure contents of the samples in each group. Compared to PEP + MUC (24 and 27%), the random crimp of DPEP + MUC (19 and 19%) decreased while the content of the other three structures increased, particularly at 12 h of interaction. Comparing the α‐helix content of polysaccharides interaction with MUC at 0 and 12 h revealed that the α‐helix content was reduced after PEP interaction with MUC for 12 h (23 and 20% as a percentage of 0 and 12 h, respectively), but the α‐helix content of DPEP was essentially unchanged after 12 h of interaction with MUC (20 and 21% as a percentage of 0 and 12 h, respectively). The interaction between polysaccharides and MUC often leads to changes in the protein's secondary structural elements (SSE) and thereby affects its stability.

## 4 CONCLUSION

The current study investigated the interaction between the *P. eryngii* polysaccharides and its digestive products with MUC. The results showed that polysaccharides can interact with MUC, and digestion can change the degree of interaction between polysaccharides and MUC. Interestingly, this trend has been enhanced time increasing within 12 h. Specifically, the tertiary structure of intertangled polysaccharides was opened after digestion, causing more binding sites, and the interaction between polysaccharides and MUC has undergone alterations. This interaction may indirectly impact the exercise and immune activities of polysaccharides and influence the transportation of other nutrients. In this paper, we initially investigate the characteristics of polysaccharides' interactions with MUC to provide valuable insight into polysaccharides entering the immune activity in the intestine.

## AUTHOR CONTRIBUTIONS

**Sai Ma:** Conceptualization (supporting); investigation (equal); writing – original draft (equal). **Xinyi Li:** Methodology (supporting); validation (supporting); writing – review and editing (supporting). **Qi Tao:** Formal analysis (supporting); software (supporting). **Qiuhui Hu:** Project administration (supporting); resources (lead). **Benard Muinde Kimatu:** Writing – review and editing (supporting). **Wenjian Yang:** Investigation (supporting); visualization (supporting). **Gaoxing Ma:** Conceptualization (equal); formal analysis (equal); funding acquisition (equal).

## FUNDING INFORMATION

This work was funded by the National Natural Science Foundation of China (grant number: 32372324), the Key Research and Development Program of Jiangsu Province (grant number: BE2022378), and the Priority Academic Program Development of Jiangsu Higher Education Institutions (grant number: PAPD).

## CONFLICT OF INTEREST STATEMENT

The authors declare no conflict of interest relevant to this article.

## ETHICS STATEMENT

The present study did not include any procedures on humans or animals.

## Supporting information


Fig. S1

## Data Availability

The data that support the findings of this study are available from the corresponding author upon reasonable request.
