# Supplementary material for: The effect of in vitro digestion on the interaction between polysaccharides derived from Pleurotus eryngii and intestinal mucus
Source: Food Sci Nutr. 2023 Nov 22;12(2):1318–29. doi: 10.1002/fsn3.3845 (PMC10867464; doi:10.1002/fsn3.3845)
Supplement: Supplementary file 1 — Fig. S1 [file FSN3-12-1318-s001.docx]

**Fig.S1** **FT-IR spectra of PEP**

**Fig.S2** **UV spectra of PEP**
